# Parameter-level comparison of artificial intelligence and manual cephalometric measurements: a systematic review and meta-analysis

**DOI:** 10.3389/fdmed.2026.1820984

**Published:** 2026-08-21

**Authors:** Franz Tito Coronel-Zubiate, Consuelo Marroquín-Soto, Joan Manuel Meza-Málaga, Sara Antonieta Luján-Valencia, Alejandro Placeres-Hernández, Angie Vanessa Carmona-Sanchez, Fredy Hugo Cruzado-Oliva, Carlos Alberto Farje-Gallardo, Jeanile Zuta-Rojas, Eduardo Luján-Urviola, Heber Isac Arbildo-Vega

**Affiliations:** 1 Faculty of Health Sciences, Stomatology School, Universidad Nacional Toribio Rodríguez de Mendoza de Amazonas, Chachapoyas, Peru; 2 Department of Dentistry, School of Dentistry, Universidad Científica del Sur, Lima, Peru; 3 Faculty of Dentistry, Dentistry School, Universidad Católica de Santa María, Arequipa, Peru; 4 Faculty of Health, Specialisation in Orthodontics and Dentofacial Orthopaedics, Universidad Autónoma de Manizales, Manizales, Colombia; 5 Faculty of Stomatology, Stomatology School, Universidad Nacional de Trujillo, Trujillo, Peru; 6 Faculty of Dentistry, Universidad Andina Néstor Cáceres Velásquez, Juliaca, Peru; 7 Faculty of Dentistry, Dentistry School, Universidad San Martín de Porres, Chiclayo, Peru; 8 Faculty of Human Medicine, Human Medicine School, Universidad San Martín de Porres, Chiclayo, Peru; 9 Posgraduate School, Universidad Nacional Toribio Rodríguez de Mendoza de Amazonas, Chachapoyas, Peru

**Keywords:** artificial intelligence, cephalometric measurements, cephalometry, meta-analysis, orthodontics

## Abstract

**Introduction:**

Prior work has largely focused on landmark-localization error and runtime; whether AI-derived parameter-level measurements differ systematically from those obtained by manual cephalometric analysis remains unclear.

**Objective:**

To compare parameter-level cephalometric measurements obtained using AI systems with manual reference methods through a systematic review and meta-analysis; secondarily, to narratively summarize any reported diagnostic metrics (κ/ICC, sensitivity/specificity, AUC) without pooling.

**Methods:**

Six databases (PubMed, Scopus, Web of Science, IEEE Xplore, EBSCO, and SciELO) were searched with no time restriction (inception to 20 September 2025) and no language limits; citation chasing was performed (Google Scholar). Eligible studies directly compared AI-based and manual cephalometric tracings. Random-effects meta-analyses were conducted only for parameter-level cephalometric measurements (primary outcomes), using standardized mean differences (Hedges’ *g*) with 95% confidence intervals (CIs). Statistical heterogeneity was quantified using the *I*^2^ statistic. Sensitivity/specificity/AUC were not meta-analyzed due to heterogeneous thresholds and ≤3 studies per metric; no bivariate or HSROC model was applied. Risk of bias was assessed with QUADAS-2 and certainty with GRADE. Legacy rule/knowledge-based reports were summarized narratively and not pooled. Registration: OSF (DOI: 10.17605/OSF.IO/WKMVD).

**Results:**

Twenty-two studies published between 2020 and 2025 were included. AI showed small and generally non-significant differences compared with manual methods across most evaluated parameters, although the certainty of evidence was low to moderate and heterogeneity was considerable for several outcomes. A statistically significant but small advantage was observed for the ANB angle (*g* = 0.28; 95% CI: 0.03–0.53; *p* = 0.03), with uncertain clinical relevance. Complementary legacy studies were described narratively and excluded from pooling. GRADE certainty ranged from low to moderate.

**Conclusions:**

AI-assisted cephalometric analysis produces parameter-level measurements comparable to manual tracings for most evaluated parameters; a small statistical difference for ANB was observed without clear clinical relevance. However, the certainty of evidence is limited by heterogeneity, publication bias, and lack of multicenter validation. AI may complement cephalometric workflows and save time, but it requires expert supervision and does not replace comprehensive orthodontic diagnosis or treatment planning. Funding/COI: As declared in the manuscript.

**Systematic Review Registration:**

https://doi.org/10.17605/OSF.IO/WKMVD.

## 1 Introduction

Manual cephalometric analysis is time-consuming and subject to inter-/intra-observer variability, which can impact diagnostic decisions and treatment planning. Orthodontic diagnosis is critical for planning treatments that correct malocclusions and optimize function and aesthetics; accuracy at this stage directly influences therapeutic decisions and outcomes. In recent years, artificial intelligence (AI) has been increasingly applied to orthodontic cephalometry, yet differences in parameter-level measurements compared with manual tracings remain uncertain (1, 2). Prior syntheses have not established whether full, clinically interpreted cephalometric parameters from AI differ from manual analysis; this review directly compares parameter-level measurements between AI-based and manual cephalometric analyses. Prior systematic reviews/meta-analyses have primarily pooled landmark-localization error and runtime; for 2D lateral cephalograms, pooled mean radial error is typically = 1.39 mm (below the 2 mm clinical threshold) with faster automated runtimes, yet with major caveats (single-center training, inconsistent “gold standards,” limited external validation, and higher/more variable error in CBCT/3D). Crucially, landmark error does not directly capture whether clinically interpreted cephalometric parameters differ from manual analysis (3, 4). Unlike prior syntheses that focused mainly on landmark-localization error, the present review evaluates full cephalometric parameters used in clinical decision-making. This addresses the unresolved gap left by landmark-error syntheses-namely, whether AI-based analyses produce parameter-level measurements that differ systematically from manual cephalometry across clinically interpreted parameters.

In dentistry, AI has supported restorative planning, caries detection, radiographic interpretation, and outcome prediction (5–7). Beyond imaging, recent studies show that large language models can answer specialty-level dental MCQs and even generate board-style items with high clarity and relevance, while still missing edge-case contraindications and high-risk details (8–10). In orthodontics, systems have been developed for automatic cephalometric landmarking, extraction-need estimation, growth assessment, and 3D aesthetic simulation (11, 12). Earlier rule-/knowledge-based approaches also explored automatic landmark identification (13); however, these systems reported per-landmark error metrics rather than clinically interpreted parameter-level measurements. Therefore, such studies are not directly comparable with contemporary deep-learning models nor with head-to-head parameter-level analyses and are cited here only for historical context. Contemporary commercial neural-network software can reduce analysis time without materially compromising clinical accuracy (14–17), although variability persists for specific parameters (18). Because small, anisotropic landmark errors may propagate non-linearly to angular and linear measurements, landmark precision alone does not guarantee parameter-level equivalence. This conceptual distinction motivates the present measurement-level synthesis focused on clinically interpreted diagnostic parameters.

These tools may expedite cephalometric measurements and reduce inter-observer variability, but require expert supervision and are not a stand-alone substitute for comprehensive orthodontic diagnosis or treatment planning. However, clinical implementation of such systems requires rigorous validation. While several primary studies have reported performance metrics such as sensitivity, specificity, area under the curve (AUC), accuracy, and concordance coefficients [kappa, ICC], most present methodological limitations, small sample sizes, heterogeneity in design, and risk of bias (2, 3, 5, 6, 19). In addition, challenges persist regarding model opacity (“black box”), algorithmic bias, data privacy, and clinical autonomy.

Although some previous reviews have addressed the role of AI in orthodontics, many have focused on technical aspects without systematically and quantitatively evaluating parameter-level comparisons between AI and orthodontists or reference standards (12–14). Even recent meta-analyses have identified notable discrepancies between AI and human clinical judgement, underscoring the need for a more focused and up-to-date synthesis (5). We prioritized routinely used, consistently reported parameters: sagittal (ANB, SNA, SNB), vertical (FMA, SN–MP), dentoalveolar (IMPA, U1–NA, L1–NB), global (interincisal angle), discrepancy (Wits), and vertical morphology (Björk–Jarabak sum), and did not pool sparsely reported or heterogeneously defined measures. In line with PRISMA 2020, our prespecified objective was to compare parameter-level cephalometric measurements obtained using AI-based systems and manual tracings across routinely used diagnostic parameters. The quantitative synthesis was designed to evaluate systematic differences between methods rather than to formally establish measurement agreement or interchangeability, which would require individual-level agreement analyses not consistently reported in the primary studies.

Accordingly, the present systematic review and meta-analysis was conducted to systematically evaluate parameter-level comparisons between AI-based cephalometric analysis and manual tracings performed by orthodontists or expert reference standards. Diagnostic performance metrics (κ/ICC, sensitivity, specificity, and AUC) were summarized narratively when reported.

This review adds value beyond prior work by (i) updating the evidence through 20 September 2025; (ii) conducting parameter-specific meta-analyses of diagnostic cephalometric measurements (e.g., ANB, SNA, SNB, IMPA), rather than focusing solely on landmark detection error; (iii) applying a GRADE framework at the outcome level; and (iv) restricting inclusion to direct head-to-head comparisons between AI-based and manual tracings. Several systematic reviews and umbrella reviews have previously addressed AI in cephalometric analysis, primarily focusing on landmark detection accuracy or location error (3, 20–22). In contrast, the most recent similar meta-analysis (18) pooled landmark-level outcomes without assessing whether such precision translates into comparable parameter-level diagnostic measurements. The present review therefore addresses a different and clinically relevant question: whether AI-derived cephalometric parameters differ systematically from those obtained by conventional manual tracings across routinely used diagnostic measurements. By synthesizing parameter-level evidence across 16 clinically relevant outcomes, incorporating additional comparative studies published in 2024–2025, and evaluating certainty using GRADE, this review extends previous evidence from technical landmark localization to clinically interpretable diagnostic measurements, thereby providing information that is more directly applicable to orthodontic diagnosis and treatment planning.

## 2 Methods

### 2.1 Design and eligibility criteria

This systematic review followed the methodological recommendations of the PRISMA 2020 statement (23). The complete checklist is provided in Supplementary Material S1. The protocol was registered in the Open Science Framework https://doi.org/10.17605/OSF.IO/WKMVD. Registration in PROSPERO was not feasible because that platform no longer accepts diagnostic accuracy reviews at the time of submission.

The research question was formulated using the PECOS framework:

- Population: patients (children, adolescents, or adults) requiring orthodontic diagnosis.
- Exposure: AI-based diagnostic methods (including machine learning and deep learning models).
- Comparator: manual cephalometric tracings performed by orthodontists or expert reference standards.
- Outcomes: Primary outcome: parameter-level cephalometric measurements (e.g., ANB, SNA, SNB, IMPA, FMA, U1–NA, L1–NB) comparing AI-based with manual tracings. Secondary (narrative-only): diagnostic performance metrics (e.g., sensitivity, specificity, AUC, accuracy, kappa/ICC) when reported; not pooled due to scale/threshold heterogeneity and sparse data.
- Study design: analytical observational studies, including comparative cross-sectional and diagnostic validation studies. Pooled synthesis was limited to parameter-level head-to-head outcomes; heterogeneous endpoints (classification tasks, landmark-error–only metrics) were retained for narrative description only and not meta-analysed.

Studies were included if they directly compared AI-based cephalometric analysis with manual tracings performed by orthodontists or expert reference standards. Reports with parameter-level head-to-head outcomes (AI-based vs. manual tracings) were eligible for meta-analysis. Legacy rule-/knowledge-based pipelines and studies reporting only per-landmark error thresholds were captured by the search and retained for narrative description but prespecified as non-combinable with parameter-level pooling due to outcome incompatibility and limited clinical applicability in the current deep-learning era. No language restrictions were applied. Exclusion criteria were systematic/narrative reviews, *in vitro* or animal studies, editorials, conference abstracts, duplicate publications, and studies without a direct AI–human/reference comparison. Preprints were considered only if no peer-reviewed version was available and sufficient methodological detail allowed risk-of-bias appraisal. Semi-automated workflows that required discretionary manual refinement of AI outputs were not pooled; only AI-attributable parameter-level results (pre-adjustment) were eligible. Records with asymmetric post-processing were retained narratively and excluded from quantitative synthesis.

### 2.2 Sources of information and search strategy

We systematically searched PubMed/MEDLINE, Scopus, Web of Science, IEEE Xplore, EBSCO, and SciELO from inception to 20 September 2025, without language restrictions. When non-English full texts were identified, they were translated into English for screening and data extraction. Full electronic strategies for each database are provided in Supplementary Material S2. Manual forward/backward citation tracking was performed up to the same cutoff date (20 September 2025); records identified after this date were logged but not included in the synthesis.

Database rationale: PubMed/MEDLINE was included for biomedical/clinical coverage; Scopus and Web of Science for broad cross-disciplinary indexing and citation tracking; IEEE Xplore for engineering/AI methods relevant to image analysis; SciELO to capture regional literature (Spanish/Portuguese); and EBSCO as an additional aggregator to reduce database-specific blind spots.

In addition, we screened reference lists of included studies and performed citation tracking. Google Scholar was used only to identify citing/related records and then de-duplicated against the core databases in Zotero; all retained Google Scholar hits were duplicates of database records or ineligible. Duplicates were removed in Zotero, and records were managed in Rayyan®.

Gray literature policy: Google Scholar served exclusively for forward/backward citation chasing; preprint servers and conference proceedings were checked at the screening stage. Conference abstracts were excluded per eligibility criteria, and preprints were retained only when no peer-reviewed version existed and sufficient methods/data were available for assessment. This approach aimed to mitigate publication bias while preserving methodological rigor. No further search updates were conducted after 20 September 2025.

To ensure comprehensive coverage of cephalometric automation approaches, the search also captured rule-/knowledge-based reports. Because these studies typically reported per-landmark error rather than parameter-level measurements, they were described narratively and not included in the quantitative synthesis [e.g., Rakhshan et al. (13), whose full text was obtained through author correspondence/ResearchGate]. No eligible non-English full-text studies were ultimately included in this review; therefore, no translations were required for eligibility assessment or data extraction.

### 2.3 Selection and data extraction

Two independent reviewers (AVC-S and CAF-G) screened all retrieved records according to the predefined eligibility criteria. Title/abstract screening was followed by full-text assessment of potentially eligible studies. Disagreements at any stage were resolved through discussion and consensus or, when necessary, by consultation with a third reviewer (EL-U). Only final consolidated screening decisions were archived; therefore, Cohen's *κ* could not be calculated retrospectively.

Data extraction was performed independently and in duplicate (JMM-M and SAL-V) using a structured Microsoft Excel matrix (Microsoft Corporation, Redmond, WA, USA) specifically designed for this review (Supplementary Material S4). Extracted information included study characteristics, participant characteristics, AI model details, comparator information, imaging modality, outcomes, and statistical methods. Any discrepancies were resolved by consensus or, when necessary, through consultation with a third reviewer (EL-U).

### 2.4 Variables collected

From each study we extracted: author, year, country, sample size, population characteristics; AI model/approach (architecture, training/validation); comparator (number/experience of orthodontists); imaging modality (2D lateral cephalogram or CBCT); diagnostic task (e.g., cephalometric analysis, skeletal pattern classification); and statistical methods.

Primary outcomes were parameter-level cephalometric measurements obtained from AI-based and manual tracings (e.g., ANB, SNA, SNB, IMPA, FMA, U1–NA, L1–NB), which were quantitatively compared using meta-analytic methods. Secondary outcomes were diagnostic performance metrics when reported (sensitivity, specificity, AUC, accuracy, kappa/ICC). We also recorded whether models underwent internal and/or external validation; diagnostic performance metrics (κ/ICC, sensitivity/specificity, AUC) were extracted when reported for narrative synthesis only.

### 2.5 Statistical analysis

Random-effects meta-analyses were conducted only for parameter-level cephalometric measurements (primary outcomes). The quantitative synthesis was designed to evaluate systematic differences between AI-derived and manual measurements rather than to formally assess measurement agreement or interchangeability, which would require individual-level agreement statistics not consistently reported by the primary studies. Although cephalometric outcomes were generally reported in common units (degrees or millimeters), substantial differences in landmark definitions, cephalometric analysis protocols, AI software, reference standards, and measurement methodologies across studies limited the direct comparability of raw mean differences. Therefore, standardized mean differences (Hedges’ *g*) were used to enable quantitative synthesis across methodologically heterogeneous studies. Heterogeneity was quantified using *I*^2^ and corresponding *p*-values. Angles were analyzed in degrees and linear distances in millimeters; no rescaling was applied across different constructs. Where studies reported SE or 95% CI, we converted to SD using standard formulae; if variance was missing and not derivable, the study was excluded from that specific meta-analysis and summarized narratively. We did not meta-analyze sensitivity/specificity/AUC because ≤3 studies per metric and non-comparable thresholds precluded valid pooling; no bivariate or HSROC model was applied. Prespecified subgroup analyses (2D vs. CBCT; AI approach; study design) and meta-regression were not conducted because no pooled outcome contained a sufficient number of studies to provide reliable estimates (minimum target ≥5 studies per subgroup and substantially more for meta-regression), and measurement definitions were insufficiently harmonized across studies. No semi-automated arms were included in the meta-analysis; studies relying on AI outputs modified by non-symmetrical human adjustments were summarized narratively only.

Exploratory assessments of small-study effects were performed using funnel plots and Egger's regression. Because all outcome-specific meta-analyses included fewer than the recommended 10 studies, these analyses were considered descriptive only and interpreted with caution rather than as formal assessments of publication bias. We did not compute a single global summary across heterogeneous endpoints; instead, parameter-specific random-effects meta-analyses were performed. Diagnostic performance metrics (κ/ICC, sensitivity, specificity, and AUC) were not meta-analyzed because of substantial differences in measurement scales, diagnostic thresholds, and the limited number of studies reporting each metric. These outcomes were therefore synthesized narratively.

### 2.6 Risk of bias assessment

The risk of bias of the included studies was assessed using the QUADAS-2 tool (24) by two independent reviewers (A.P.-H., F.C.-O.), with a third reviewer (C.M.-S.) resolving disagreements. QUADAS-2 domains included patient selection, index test, reference standard, and flow/timing, plus applicability concerns. We specifically considered potential bias from asymmetric manual refinement in semi-automated workflows (index-test/applicability). Outcome-level GRADE downgrades were based on domain-level QUADAS-2 judgments (not a single “overall” score) for risk of bias, alongside inconsistency, imprecision, indirectness, and publication bias, as detailed in Supplementary Material S5. Detailed outcome-level certainty assessments were performed separately for each pooled cephalometric parameter using the GRADE approach (51). These assessments considered risk of bias (derived from QUADAS-2 domain judgments), inconsistency, indirectness, imprecision, and exploratory publication bias. Complete evidence profiles for all pooled outcomes are provided in Supplementary Material S8. Only final consolidated QUADAS-2 judgments were archived; therefore, Cohen's *κ* could not be calculated retrospectively. When assessing the Reference Standard domain of QUADAS-2, particular attention was paid to the description of examiner expertise, calibration procedures, repeatability, and construction of the manual reference standard. Studies with insufficient reporting of these aspects were judged as having increased uncertainty or risk of bias in the reference standard domain.

## 3 Results

### 3.1 Study selection

The systematic search covered six electronic databases: PubMed/MEDLINE, Scopus, Web of Science, EBSCO, IEEE Xplore, and SciELO, with no date limits (from inception) and was updated to 20 September 2025. Search strategies combined controlled vocabulary and free-text keywords related to artificial intelligence, orthodontics, diagnosis, and measurement agreement (sensitivity, specificity, AUC, agreement). Records identified after the prespecified cutoff date were logged but not included in the synthesis. Google Scholar was used solely for forward/backward citation identification, and all hits were de-duplicated against core databases to ensure reproducibility and minimize inclusion bias.

**Figure 1:**
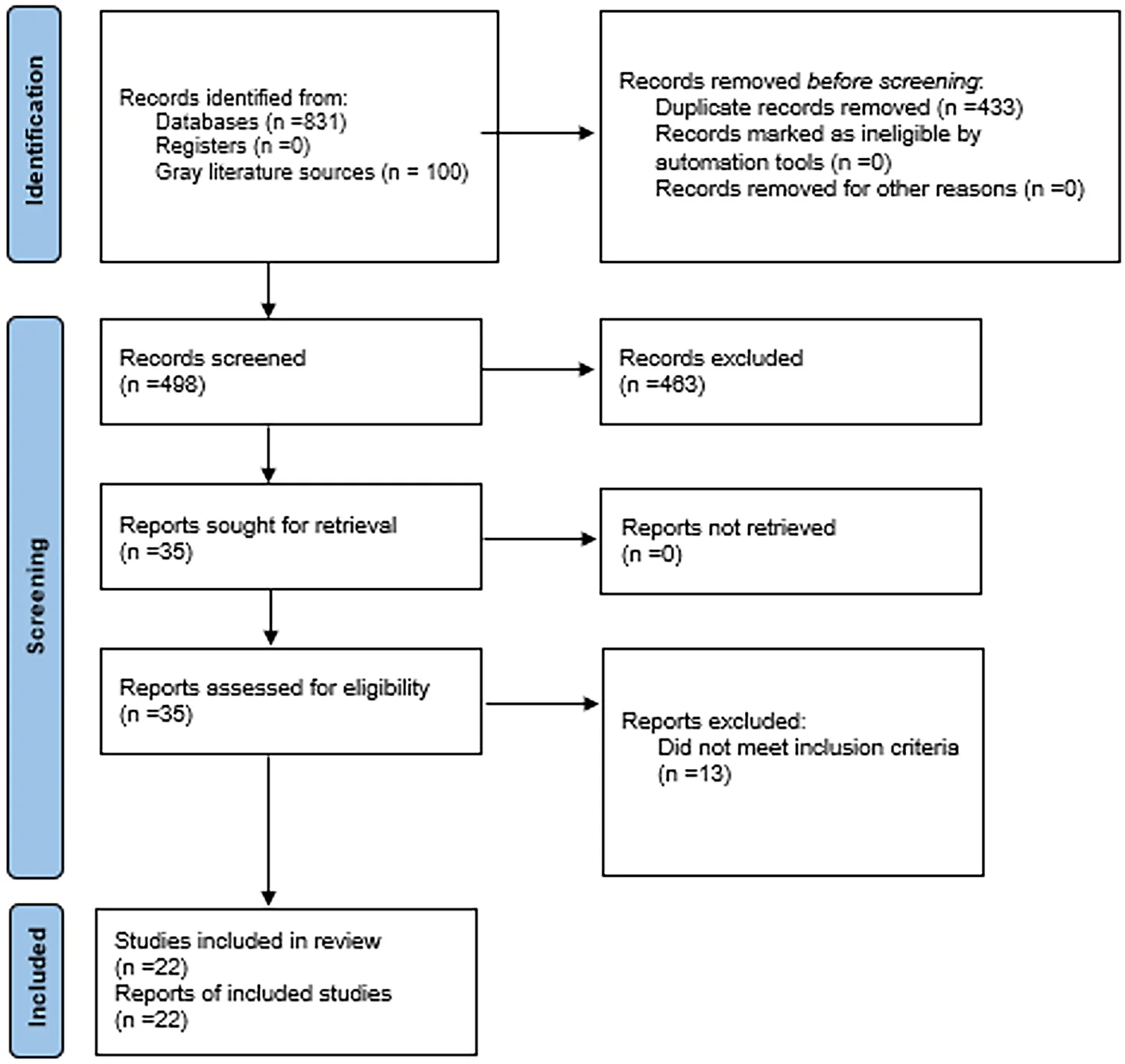
PRISMA 2020 flow diagram. Databases: PubMed/MEDLINE, Scopus, Web of Science, EBSCO, SciELO, IEEE Xplore. Google Scholar was used only for forward/backward citation chasing and was de-duplicated against core databases. Totals: identified *n* = 931; duplicates *n* = 433; screened *n* = 498; full-text assessed *n* = 35; excluded with reasons *n* = 13; included *n* = 22. Legacy rule-/knowledge-based reports (Rakhshan 2009) were recorded narratively only and not pooled due to outcome incompatibility.

A total of 303 records were identified in PubMed, 260 in Scopus, 173 in Web of Science, 40 in IEEE Xplore, 51 in EBSCO, and 4 in SciELO. Additionally, 100 records were retrieved from Google Scholar during citation chasing (all already represented in the core databases or ineligible). In total, 931 records were identified. After removing 433 duplicates (Zotero), 498 titles/abstracts were screened; 35 full-text articles were assessed for eligibility, and 13 were excluded with reasons (Supplementary Material S3 and Figure 1).

Title/abstract and full-text screening were performed independently by two reviewers, with disagreements resolved by consensus or, when necessary, adjudication by a third reviewer. The most frequent reasons for exclusion at full text were: absence of a manual orthodontist/reference comparator; reporting only landmark-error metrics without parameter-level outcomes; insufficient or non-extractable variance data for pooling; asymmetric semi-automated workflows (AI outputs manually refined in a non-comparable way); non-cephalometric imaging/tasks; or conference abstracts/reviews/duplicates.

Ultimately, 22 studies (4, 16, 19, 25–43) met the inclusion criteria and were incorporated into the qualitative and quantitative synthesis (Supplementary Material S6). Complementary rule-/knowledge-based reports [e.g., (13)] were described narratively and not pooled due to outcome incompatibility with parameter-level head-to-head analyses.

Funding sources and conflicts of interest were also extracted and are summarised in Supplementary Material S6.

### 3.2 Characteristics of the included studies

The general characteristics of the included studies are summarized in Supplementary Material S4 (Data Extraction Matrix). The included studies were published between 2020 and 2025 and were conducted in diverse geographic settings, including the United States, South Korea, Brazil, Germany, Spain, Turkey, China, Saudi Arabia, Pakistan, Nepal, Belgium, Russia, and India (19, 25–43). Most studies were single-centre, observational diagnostic comparisons using 2D lateral cephalograms or CBCT (19, 25–43); AI systems included both commercial and bespoke models, including conventional machine-learning and deep-learning approaches, vs. manual tracings performed by expert orthodontists or experienced residents (19, 25–43). Parameter-level cephalometric measurements were the primary pooled outcomes; Diagnostic metrics (κ/ICC, sensitivity/specificity, AUC) were variably reported and are summarized narratively (19, 25–43). Where semi-automated pipelines were identified, they did not meet pooling criteria (AI-only or symmetrically adjusted outputs) and were therefore not meta-analyzed.

Interventions involved various AI platforms for cephalometric analysis (WebCeph, CephX, DentaliQ.ortho, AudaxCeph, RadioCef, NemoCeph) and bespoke systems using conventional machine learning or convolutional neural networks (CNNs) (16, 17, 19, 25–43). In most cases, the comparators were manual tracings performed by expert orthodontists or experienced residents, considered the reference standard (19, 25–43).

The most frequently reported agreement/accuracy metrics were the intraclass correlation coefficient (ICC) and mean absolute error (MAE), and, less frequently, AUC and Lin's concordance correlation coefficient (CCC) (19, 25–43). The primary outcome for pooling was the comparison of parameter-level cephalometric measurements obtained from AI-based and manual tracings. Diagnostic performance metrics were summarized qualitatively when reported. Reliability reporting was heterogeneous (methods and indices varied), precluding pooled synthesis.

### 3.3 Risk of bias assessment

Supplementary Material S5 summarizes the risk of bias assessment performed using the QUADAS-2 tool. Risk-of-bias assessments were conducted independently by two reviewers, with disagreements resolved by consensus or, when necessary, by consultation with a third reviewer. Inter-reviewer agreement (Cohen's *κ*) was not calculated because only the final consensus judgments were retained.

In the Patient Selection domain, 15 studies were judged to have a low risk of bias (4, 19, 25, 28, 29, 31–36, 39, 40), five an unclear risk (16, 27, 37, 38, 43), and two a high risk (30, 41). In the Index Test domain, 15 studies were classified as low risk (4, 16, 19, 27–29, 31–36, 39, 40, 42, 43), whereas seven were judged as having unclear risk (25, 26, 30, 37, 38, 41, 43). In the Reference Standard domain, 18 studies were considered low risk (4, 16, 19, 25, 26, 28, 29, 31–35, 38–40, 42, 43), and four were classified as high risk (27, 30, 37, 41). The Flow and Timing domain showed predominantly low risk of bias (4, 16, 19, 25–29, 31–36, 38–42), with only two studies judged as having unclear risk because of insufficient reporting (37, 43).

Across several studies, manual tracings were performed by a single examiner or by residents without clearly reported calibration procedures, and examiner experience was often insufficiently described. These limitations may have affected the reliability of the reference standard and contributed to uncertainty in AI–manual comparisons.

Applicability concerns were evaluated separately for the Patient Selection, Index Test, and Reference Standard domains, following the QUADAS-2 framework (Supplementary Material S5). Most studies showed low concern regarding applicability for at least one domain, whereas higher or unclear concerns were mainly associated with highly selected study populations, limited clinical settings, or insufficient reporting of the index test or reference standard, which may restrict the generalizability of the findings.

Overall, most studies demonstrated low risk of bias in the Patient Selection and Index Test domains. However, concerns were more frequent in the Reference Standard domain because manual tracings performed by orthodontists or residents were not consistently defined or validated as the reference standard. In addition, incomplete reporting of examiner calibration, blinding procedures, and measurement repeatability contributed to uncertainty in several studies. These methodological limitations were incorporated into the outcome-level GRADE assessment, together with inconsistency and imprecision.

### 3.4 Quantitative analysis

We conducted outcome-specific meta-analyses for 16 cephalometric measurements. Supplementary Material S7 contains the complete statistical outputs for each parameter-specific meta-analysis, including the corresponding forest plots, funnel plots, and exploratory Egger's regression results. For outcomes supported by ≤2–3 studies and/or with wide CIs, we report the result as inconclusive due to limited data and imprecision, rather than as “no difference”. Prespecified subgroup analyses (2D vs. CBCT; AI approach; study design) were not feasible because no outcome met the minimum data threshold per subgroup and measurement definitions were not sufficiently harmonized across studies. Exploratory funnel plots and Egger's regression were performed for all outcome-specific meta-analyses. Because all analyses included fewer than the recommended 10 studies, these results were considered descriptive only and interpreted cautiously rather than as formal assessments of publication bias.

Concise summary of pooled outcomes: Across the 16 parameter-specific meta-analyses, most skeletal and dentoalveolar measurements showed small pooled effects with confidence intervals including the null, indicating inconclusive or minimal differences between AI and manual tracings (SNA, SNB, IMPA, interincisal angle, U1–SN, L1–MeGo, Wits, gonial angle). A small statistically significant difference favoring AI was observed only for ANB with a small effect (*g* = 0.28; 95% CI: 0.03–0.53), of uncertain clinical relevance. Inconclusive results—due to limited studies and/or wide CIs—were seen for U1–NA (angle/distance), L1–NB (angle/distance), FMA, SN–MP, and SUM–MAN.

Multiplicity: Given multiple, correlated outcomes, no formal multiplicity adjustment was applied; Interpretation emphasizes effect sizes and 95% CIs together with GRADE certainty rather than isolated *p*-values. Accordingly, the single statistically significant finding (ANB) is interpreted cautiously with low certainty for clinical impact.

Bias and heterogeneity interpretation: Heterogeneity was low to moderate for several sagittal/dentoalveolar measures, but substantial for vertical/global parameters (FMA *I*^2^ = 83%, SUM–MAN *I*^2^ = 94%), prompting downgrades for inconsistency in GRADE. Exploratory analyses of small-study effects should be interpreted cautiously because all meta-analyses included fewer than the recommended 10 studies. According to Supplementary Material S7, most outcomes showed no evidence of funnel plot asymmetry, although isolated statistically significant Egger's test results were observed and should be interpreted cautiously given the limited number of included studies. Frequent reference-standard concerns (inconsistent definition of the manual “gold standard”) also contributed to risk-of-bias downgrades.

Key findings: ANB showed a small but statistically significant difference favoring AI (*g* = 0.28; 95% CI: 0.03–0.53) with uncertain clinical relevance. Vertical measurements (e.g., FMA) exhibited substantial heterogeneity and yielded inconclusive pooled effects. Most other skeletal/dental parameters (e.g., SNA, SNB, IMPA, interincisal angle, U1–SN, L1–MeGo, Wits, gonial angle, SUM–MAN) were inconclusive due to limited data and/or wide CIs. Certainty for underpowered/highly heterogeneous outcomes was downgraded in GRADE for inconsistency and imprecision.

For the U1–NA angle (°), two studies were included (30, 35), evidence was inconclusive due to limited data and imprecision (*g* = −0.11; 95% CI: −0.50 to 0.39; *p* = 0.67). Similarly, the U1–NA distance (mm) (4, 39), evidence was inconclusive owing to limited data and wide CIs (*g* = −0.29; 95% CI: −1.01 to 0.44; *p* = 0.44).

The L1–NB angle (°) (30, 35) no statistically conclusive differences were identified given the small evidence and imprecision (*g* = −0.22; 95% CI: −0.96 to 0.53; *p* = 0.57). Regarding the L1–NB distance (mm) (4, 39), the result was inconclusive (*g* = 0.29; 95% CI: −0.08 to 0.63; *p* = 0.10) due to limited data and CIs spanning benefit and harm.

IMPA (°) (34, 39), evidence was inconclusive given the very small number of studies and narrow clinical effects (*g* = −0.03; 95% CI: −0.28 to 0.22; *p* = 0.80). For the U1–SN angle (°) (4, 29, 34, 39), the pooled effect was small and not conclusive, with CIs including the null (*g* = −0.18; 95% CI: −0.38 to 0.01; *p* = 0.07).

The interincisal angle (°) (4, 34, 40), evidence was inconclusive due to limited data and inaccuracy (*g* = 0.19; 95% CI: −0.04 to 0.42; *p* = 0.10). For L1–MeGo (°) or the mandibular incisor plane angle (4, 29, 30, 40), the pooled effect was small with CIs spanning the null, yielding inconclusive evidence of differences (*g* = −0.12; 95% CI: −0.40 to 0.15; *p* = 0.38).

For SNA angle (°) (4, 29, 30, 34, 38, 40), no consistent difference was detected; the pooled effect was small with CIs including the null (*g* = 0.11; 95% CI: −0.03 to 0.24; *p* = 0.14). Similar results were obtained for the SNB angle, involving the same studies, no consistent difference was observed (*g* = −0.12; 95% CI: −0.28 to 0.04; *p* = 0.13).

For FMA angle (°) (4, 34, 39, 40), there was a suggestive reduction in the AI group but the result was not conclusive due to substantial heterogeneity and imprecision (*g* = −0.47; 95% CI: −0.99 to 0.05; *p* = 0.08). The SN–MP (°) or SN–MeGo (°) (29, 34), evidence was inconclusive given the very limited data (*g* = −0.03; 95% CI: −0.34 to 0.28; *p* = 0.86).

In the ANB angle (°), six studies (25, 29–31, 35, 36) reported a statistically significant difference in favor of AI (*g* = 0.28; 95% CI: 0.03 to 0.53; *p* = 0.03). Given that ANB is expressed in degrees, a small standardized effect may correspond to a sub-degree difference in many clinical contexts and may not alter skeletal classification. Therefore, this finding should not be interpreted as evidence of measurement interchangeability between AI and manual tracings. The Wits appraisal (mm) (4, 34, 40), evidence was inconclusive due to limited data and imprecision (*g* = 0.14; 95% CI: −0.10 to 0.38; *p* = 0.25).

For the Gonial angle (°) (4, 40) evidence was inconclusive given the small evidence base and wide CIs (*g* = −0.59; 95% CI: −1.90 to 0.73; *p* = 0.38). Finally, the SUM–MAN (°) or Björk–Jarabak Sum included three studies (4, 34, 40), evidence was inconclusive with high heterogeneity and imprecision (*g* = 0.55; 95% CI: −0.52 to 1.61; *p* = 0.31).

The heterogeneity analysis indicated variable *I*^2^ values, with no heterogeneity in several outcomes (IMPA, U1–SN, SNA, Wits, Gonial) and high levels in others such as FMA (82.56%) and SUM–MAN (93.85%). Exploratory Egger's regression analyses showed no evidence of funnel plot asymmetry for most outcomes. Because all outcome-specific meta-analyses included fewer than the recommended 10 studies, isolated statistically significant results should be interpreted cautiously and not as conclusive evidence of publication bias. Complete Egger's regression results are presented in Supplementary Material S7.

Accordingly, certainty for underpowered or highly heterogeneous outcomes was downgraded in GRADE (inconsistency/imprecision), as detailed in Supplementary Material S8. A concise overview of the pooled quantitative findings is presented in Supplementary Material S9, whereas the corresponding outcome-level certainty of evidence (GRADE) is summarized in Supplementary Material S10.

Subgroup pooling (2D vs. CBCT; conventional vs. deep learning) was not performed because <3 studies per stratum were available; exploratory differences, where noted, are described narratively.

## 4 Discussion

The aim of this systematic review with meta-analysis was to compare parameter-level cephalometric measurements obtained using AI systems and manual methods performed by orthodontists or expert reference standards. For most angular and linear measurements, pooled effects were small and confidence intervals included the null, indicating no clinically meaningful differences between AI and manual tracing. A small statistical advantage was observed for ANB; however, its clinical impact remains uncertain and is unlikely to change diagnostic classification or treatment planning in routine practice. In day-to-day workflows, AI can accelerate measurement acquisition under specialist oversight, whereas the final diagnostic synthesis and treatment decisions remain clinician-led.

### 4.1 What this review adds

Unlike prior syntheses that primarily pooled landmark-localization error and runtime, this review meta-analyses parameter-level cephalometric measurements (ANB, SNA, SNB, IMPA) in direct head-to-head comparisons between AI systems and manual tracings, providing a parameter-level comparative perspective that is closer to clinical decision-making. We also applied outcome-specific GRADE to quantify certainty, explicitly separated non-combinable legacy/landmark-only reports to avoid construct mixing, and mapped where heterogeneity and imprecision undermine certainty (notably vertical measurements). This framework clarifies where AI-derived measurements show small average differences relative to manual tracings and where the available evidence remains uncertain.

### 4.2 These findings are consistent with prior literature

A recent systematic review (18) reported ICC above 0.90 for most landmarks using convolutional neural networks, although inter-study variability persisted due to heterogeneity in datasets and methodologies. Katyal et al. (4) primarily pooled landmark-localization error and showed sub-millimeter accuracy, but did not evaluate whether this technical performance translated into comparable evaluate clinically interpreted cephalometric parameters. Our review addresses a different clinical question by evaluating whether this technical precision translates into comparable clinically interpreted cephalometric parameters, showing small and clinically non-meaningful AI–manual differences for most measures. Hendrickx et al. (3) similarly reported high agreement (ICCs >0.90) and no consistent differences in key parameters; our meta-analyses corroborate that pattern while additionally providing outcome-specific certainty assessments using GRADE and identifying where evidence remains limited (particularly for some vertical/global measurements). In line with advances in AI models, Qian et al. (44) introduced CephaNN, a multi-head attention network that reduced MAE in difficult landmarks, yet improvements in landmark localization did not necessarily translate into clinically meaningful differences in cephalometric measurements. Likewise, Bichu et al. (45) noted that most AI studies rely on limited datasets and lack external validation, a limitation also observed in the primary studies included in our review. Earlier rule-/knowledge-based pipelines (13) further illustrate that acceptable per-landmark error does not necessarily guarantee comparable parameter-level measurements, reinforcing the rationale for the present synthesis.

Beyond individual studies, several systematic and umbrella reviews have examined AI in cephalometry. A systematic review synthesized available evidence and concluded that AI achieved acceptable accuracy for landmark detection, but did not evaluate parameter-level diagnostic measurements (20). A 2022 meta-analysis by Katyal et al. similarly focused on landmarking precision across 13 studies, reporting sub-millimetre error rates but without assessing their impact on clinically used cephalometric parameters (4). A further umbrella review addressed automatic landmark identification but did not provide parameter-level meta-analyses (22). Similarly, Serafin et al. reported that deep-learning algorithms achieved promising accuracy for automated three-dimensional cephalometric landmark identification, although substantial heterogeneity and methodological variability limited confidence in pooled estimates and highlighted the need for standardized protocols and multicenter validation (46). In contrast, the present review not only updates the evidence through 20 September 2025 (with no additional eligible studies identified between 12 June and 20 September 2025) but also conducts outcome-specific meta-analyses of diagnostic measurements (ANB, SNA, SNB, IMPA), applies the GRADE framework to assess certainty, and restricts inclusion to head-to-head comparisons with manual tracings. By comparing parameter-level cephalometric measurements, this review complements previous landmark-based syntheses by evaluating whether AI performance translates into comparable clinically interpreted diagnostic measurements. This clinically oriented perspective was not directly addressed in previous systematic reviews and meta-analyses. In addition, complementary legacy rule-/knowledge-based reports (13) were retained narratively and not pooled due to outcome incompatibility with parameter-level head-to-head analyses.

Francisco et al. (47) reviewed the current state of three-dimensional digital technologies integrated with AI in orthodontics, highlighting their potential to optimise diagnosis and planning by combining CBCT, intraoral scanning, and CAD/CAM software. While promising, none of the studies in this synthesis implemented a fully integrated workflow, representing an important avenue for future research.

The results of this review, showing generally small average differences or inconclusive findings across most measurements, are consistent with Hendrickx et al. (3), who reported ICCs above 0.90 for most cephalometric points and no significant differences in key parameters. Similarly, Mesquita et al. (48) found high agreement for SNA, SNB, and ANB, confirming that statistical equivalence does not necessarily imply clinical advantage. The slight superiority of AI for ANB observed here aligns with Hung et al. (49), who noted that AI performance varies by algorithm type and image resolution, with improvements in sagittal parameters but variability in vertical and dentoalveolar measurements. The ANB angle was the only parameter with a statistically significant difference favouring AI; however, the effect size was small and its clinical relevance remains uncertain.

Khanagar et al. (50) emphasised that, despite the high statistical agreement observed across multiple studies, the lack of multicentre validation and dataset heterogeneity limit extrapolation to other contexts. This aligns with our findings, where most included studies were single-centre and externally unvalidated.

Current evidence suggests that AI-derived parameter-level measurements generally show only small average differences relative to conventional manual tracings across most evaluated outcomes. However, formal measurement agreement or interchangeability cannot be established from the available evidence because the pooled estimates were derived from standardized mean differences rather than individual-level agreement analyses. Furthermore, although the pooled effect for the ANB angle reached statistical significance (Hedges’ *g* = 0.28; 95% CI: 0.03–0.53), the observed effect size was small and should not be interpreted as evidence of clinically meaningful disagreement or measurement interchangeability. Because standardized mean differences are unitless, the clinical relevance of this finding cannot be directly inferred from the pooled estimate and should instead be confirmed through studies reporting individual-level agreement statistics together with predefined clinical equivalence thresholds. Furthermore, many pooled estimates were derived from only a small number of studies; Therefore, these findings should be interpreted as preliminary evidence and should not be considered sufficient to support changes in clinical decision-making or treatment planning. Notably, vertical measurements (FMA and SUM–MAN) exhibited higher heterogeneity, which, together with wide confidence intervals, reduced the certainty of evidence for these outcomes. Consequently, current evidence supports the use of AI primarily as a tool to improve the efficiency of cephalometric measurement acquisition under professional supervision, while comprehensive orthodontic diagnosis, treatment planning, and clinical decision-making should remain clinician-led. Importantly, although the observed average differences were generally small, the available evidence does not establish whether these numerical differences influence diagnostic classification, treatment planning, therapeutic decisions, or patient outcomes. These clinically meaningful endpoints should therefore be prioritized in future prospective validation studies.

Because these outcomes were supported by only three to four primary studies, formal investigations of heterogeneity through subgroup analyses or meta-regression were not methodologically appropriate. The observed heterogeneity most likely reflects differences in imaging modalities, cephalometric protocols, landmark definitions, AI architectures, reference standards, examiner calibration, and study populations, although the limited number of studies precluded reliable quantitative exploration of these potential sources.

Schwendicke et al. (6) similarly noted that AI systems in diagnostic dentistry can match expert clinicians but require standardised protocols, diverse training datasets, and multicentre validation. Complementarily, Junaid et al. (20) showed accuracy varies by anatomical location, higher in sagittal points, lower in vertical or soft-tissue references, explaining discrepancies between studies and the absence of consistent AI superiority. A recent evaluation (21) also found that while AI reduces processing time, clinical utility is maximised when professionals manually review and adjust generated coordinates, supporting a hybrid approach.

AI appears feasible as a complementary tool in cephalometric analysis, expediting measurement workflows under professional oversight.

From an evidence-certainty perspective, by shifting the unit of analysis from landmark error to clinically interpreted parameters, this review delineates when small statistical differences are unlikely to alter diagnosis (most measurements) and where uncertainty persists (vertical measures with high heterogeneity), translating prior technical performance into clinically meaningful inference. Importantly, these findings should be interpreted in the context of substantial methodological and clinical heterogeneity across the included studies, including differences in AI architectures, commercial vs. custom-developed software, imaging modalities (2D vs. CBCT), landmark definitions, acquisition protocols, reference standards, and study populations. Although random-effects models and outcome-level GRADE assessments were used to account for this variability, such heterogeneity inevitably reduces confidence in the pooled estimates and limits their generalizability across clinical settings. We emphasise effect sizes and 95% CIs over isolated *p*-values; statistical significance does not imply clinical relevance, and, given multiple correlated outcomes, the single significant ANB result is interpreted cautiously with low certainty for clinical impact (per GRADE). Substantial heterogeneity was observed for FMA and SUM–MAN, which limits confidence in these pooled estimates. In addition, potential publication bias was detected for U1–NA (mm) and SUM–MAN, suggesting that the true effect may differ from the reported results. Accordingly, certainty for underpowered or highly heterogeneous outcomes was downgraded in GRADE (inconsistency/imprecision), as detailed in Supplementary Material S8.

This review has some limitations that should be acknowledged. First, the number of primary studies available for certain cephalometric parameters was limited, with some meta-analyses based on only two or three studies, which restricts the robustness of pooled estimates. Of the 16 parameter-specific meta-analyses, 10 were based on ≤3 primary studies (U1–NA angle, U1–NA distance, L1–NB angle, L1–NB distance, IMPA, U1–SN, SN–MP, interincisal angle, Wits, and SUM–MAN), which inherently limits precision and increases the risk of unstable pooled estimates. Second, most included studies were single-center and lacked external multicenter validation, reducing the generalizability of findings. Moreover, the limited number of studies available for most outcomes precluded meaningful subgroup analyses according to imaging modality, AI architecture, software platform, or patient characteristics. Consequently, potentially important sources of between-study heterogeneity could not be explored and should be addressed in future research. Third, substantial methodological and clinical heterogeneity existed across the included studies. This heterogeneity arose from differences in AI architectures, commercial vs. custom-developed software, imaging modalities (2D lateral cephalograms vs. CBCT), acquisition protocols, landmark definitions, reference standards, and study populations. Potential confounding factors likely contributed to between-study heterogeneity and imprecision, including differences in radiograph modality and acquisition parameters (2D vs. CBCT, resolution, field of view), landmark definitions and tracing conventions, examiner experience and calibration, AI architecture and training datasets, semi-automated workflows, and population characteristics (age, malocclusion severity, ethnicity). Together, these factors may have inflated or attenuated AI–manual differences, thereby reducing confidence in the pooled estimates and limiting their generalizability across clinical settings. Because the included studies differed in cephalometric protocols, landmark definitions, AI software, and reference standards, standardized mean differences were used instead of raw mean differences. Although this approach allowed quantitative synthesis across heterogeneous methodologies, standardized effect sizes are less directly interpretable in clinical units (degrees or millimeters), and this should be considered when interpreting the magnitude of pooled effects. Consequently, the pooled estimates should be interpreted as average effects across heterogeneous study settings rather than universally applicable estimates. Fourth, although the GRADE framework was applied, the certainty of evidence was often downgraded due to inconsistency, imprecision, and risk of bias; these reasons were explicitly detailed in the Supplementary Material. Fifth, although manual tracings performed by orthodontists or experienced residents were generally considered the reference standard, the quality and consistency of this comparator varied considerably across studies. In several studies, examiner experience, calibration procedures, and intra- or inter-examiner reliability were insufficiently reported, making it difficult to distinguish true AI–manual differences from variability within the reference standard itself. Consequently, uncertainty in the reference standard reduces confidence in the pooled estimates and their interpretation. Sixth, earlier rule/knowledge-based cephalometry reports (13) used per-landmark error outcomes that are not directly comparable with parameter-level head-to-head analyses and were therefore retained narratively and not pooled. Finally, the systematic search was updated to 20 September 2025 (with no additional eligible studies identified after 12 June 2025); Consequently, studies published after this search update were not included and should be considered in future updates. Taken together, these limitations indicate that the overall certainty of the body of evidence remains low to moderate. Accordingly, the findings of this review should be interpreted with considerable caution, particularly for outcomes supported by few studies, substantial heterogeneity, or low-certainty evidence, until further multicenter validation studies using standardized methodologies become available.

### 4.3 Limitations of the review process

Screening, data extraction, and QUADAS-2 assessments were conducted by two independent reviewers with consensus or third-reviewer adjudication; only consolidated decisions were archived, so inter-reviewer *κ* could not be computed retrospectively. Automated tools were used only for workflow support, Zotero for de-duplication and Rayyan® for screening, both followed by manual verification to reduce the chance of missed or wrongly excluded records. No *post hoc* modifications were made to eligibility criteria or the statistical plan after protocol registration; the decision to pool only parameter-level measurements and to narratively summarize heterogeneous diagnostic metrics (κ/ICC, sensitivity/specificity, AUC) was prespecified.

Although manual cephalometric tracings performed by orthodontists or residents are widely accepted as the reference standard, this comparator is not exempt from bias. Variability in expertise, intra- and inter-examiner reliability, and subjective judgment may influence landmark identification and measurements. These factors may have contributed to discrepancies observed across studies and should be considered when interpreting AI–manual comparisons. Consequently, external validity remains fragile, and results should be generalized with caution until robust multicenter validation is available.

### 4.4 Implications for practice

AI-assisted cephalometric analysis is feasible as a supportive adjunct that can expedite measurement acquisition while producing parameter-level measurements showing generally small average differences compared with manual tracings for most parameters under expert supervision. Given the small, statistically significant ANB difference of uncertain clinical relevance, AI outputs should be reviewed by orthodontists and integrated into a clinician-led diagnostic synthesis—not used as a stand-alone decision tool.

### 4.5 Implications for policy and education

Safe adoption would benefit from institutional guidance and curricular integration, including structured training on AI limitations (e.g., higher heterogeneity in vertical/global measures), procedures for human oversight and quality control, and minimum reporting standards (dataset description, internal/external validation, and audit trails) to support transparent evaluation, procurement, and periodic re-credentialing of AI tools.

### 4.6 Implications for future research

Priorities include prospective studies evaluating whether agreement in parameter-level cephalometric measurements translates into comparable diagnostic classifications, treatment planning decisions, therapeutic management, and patient outcomes, together with standardized benchmarking datasets and external multicenter validation. Methodologically, studies should harmonize parameter definitions, report variance data consistently, preregister protocols, and consider explainable AI (XAI) and open code/models to improve interpretability, reproducibility, and clinical trust.

### 4.7 Data governance, ethics, and transparency

Deployment should follow privacy-by-design principles with robust data governance (consent, de-identification, access control) and auditability of model versions and manual edits. Developers and investigators should disclose training data sources, bias assessments, and model update logs, and implement monitoring for performance drift across populations.

### 4.7 Economic and time-efficiency considerations

Although formal cost-effectiveness was not assessed, existing reports indicate time savings in measurement workflows. Future evaluations should quantify time, cost, and throughput gains against licensing, maintenance, and oversight costs, including the value of clinical time reallocation and downstream effects on care pathways.

## 5 Conclusions

Current evidence suggests that AI systems generally yield parameter-level cephalometric measurements showing only small average differences relative to manual tracings across most evaluated parameters. However, these findings should not be interpreted as evidence of formal measurement agreement or interchangeability because the available primary studies did not consistently report individual-level agreement statistics. Although the pooled estimate for the ANB angle reached statistical significance, it represented a small standardized mean difference and should therefore be interpreted cautiously. Its clinical significance remains uncertain because the available evidence does not allow assessment of measurement agreement or clinical equivalence. Taken together, these comparative findings support AI as a complementary tool rather than a substitute for expert assessment, although the low-to-moderate certainty of the evidence does not justify changes to current clinical decision-making pathways. Clinically, AI may facilitate cephalometric measurement acquisition and potentially reduce routine variability, provided that outputs are reviewed and, when necessary, corrected by clinicians. However, current evidence remains insufficient to support autonomous diagnostic use or modifications to orthodontic treatment planning. Certainty was limited by between-study heterogeneity (particularly for some vertical measurements), potential publication bias, and the lack of multicenter external validation. Future research should prioritize multicenter external validation across diverse populations, harmonized parameter definitions to facilitate quantitative synthesis, and routine reporting of individual-level agreement statistics (e.g., Bland–Altman analyses, limits of agreement, or intraclass correlation coefficients), together with parameter-level measurements and diagnostic performance metrics (κ/ICC, sensitivity, specificity, and AUC). Establishing shared benchmarking datasets, preregistered protocols, and transparent data and code sharing will improve reproducibility and facilitate the safe clinical translation of AI-assisted cephalometric analysis.

## Data Availability

The original contributions presented in the study are included in the article/Supplementary Material, further inquiries can be directed to the corresponding author.
