# Supplementary material for: Parameter-level comparison of artificial intelligence and manual cephalometric measurements: a systematic review and meta-analysis
Source: Front Dent Med. 2026 Aug 21;7:1820984. doi: 10.3389/fdmed.2026.1820984 (PMC13541505; doi:10.3389/fdmed.2026.1820984)
Supplement: Supplementary file 1 [file Supplementaryfile1.docx]

Supplementary Material 1: Search Strategies for Each Database

*Initial database searches were run on 12 June 2025. A top-up search using the same strategies was performed on 20 September 2025; no additional eligible studies were identified at full text. No language limits were applied. Google Scholar was used for forward/backward citation identification only and was fully de-duplicated against core databases.*

**Table 1. Bibliographic search strategy in PubMed**

| **Database** | **PubMed** ([**https: www//pubmed.ncbi.nlm.nih.gov**](https://pubmed.ncbi.nlm.nih.gov))  **Search date:** Inception - September 20, 2025 | | **Results** |
| --- | --- | --- | --- |
| **Strategy** | **#1** | ("Artificial Intelligence"[MeSH Terms] OR "Machine Learning"[MeSH Terms] OR "Deep Learning"[MeSH Terms] OR "Artificial Intelligence"[Title/Abstract] OR "Machine Learning"[Title/Abstract] OR "Deep Learning"[Title/Abstract]) | 385.373 |
|  | **#2** | ("Orthodontics"[MeSH Terms] OR "Malocclusion"[MeSH Terms] OR "Cephalometry"[MeSH Terms] OR "Orthodontics"[Title/Abstract] OR "Malocclusion"[Title/Abstract] OR "Cephalometry"[Title/Abstract]) | 102.682 |
|  | **#3** | ("Diagnosis"[MeSH Terms] OR "Diagnosis, Computer-Assisted"[MeSH Terms] OR "Diagnosis"[Title/Abstract] OR "Cephalometric"[Title/Abstract]) | 10.930.960 |
|  | **#4** | ("Sensitivity and Specificity"[MeSH Terms] OR "Area Under Curve"[MeSH Terms] OR "Observer Variation"[MeSH Terms] OR "Accuracy"[Title/Abstract] OR "Agreement"[Title/Abstract] OR "Concordance"[Title/Abstract]) | 1.646.818 |
|  | **#5** | #1 and #2 and #3 and #4 | **303** |

**Table 2. Bibliographic search strategy in SCOPUS**

| **Database** | **SCOPUS (** <https://scopus.uamanizales.elogim.com/pages/home#basic> )  **Search date:** Inception - September 20, 2025 | | **Results** |
| --- | --- | --- | --- |
| **Strategy** | **#1** | TITLE-ABS-KEY ("Artificial Intelligence" OR "Machine Learning" OR "Deep Learning") | 6.278.028 |
|  | **#2** | TITLE-ABS-KEY ("Orthodontics" OR "Orthodontic Treatment" OR "Malocclusion" OR "Dental Malocclusion" OR "Cephalometry" OR "Craniofacial Measurements") | 234.146 |
|  | **#3** | TITLE-ABS-KEY ("Diagnosis" OR "Computer-Assisted Diagnosis" OR "Cephalometric Analysis" OR "Cephalometric Measurements") | 9.145.304 |
|  | **#4** | " TITLE-ABS-KEY ("Sensitivity and Specificity" OR "Diagnostic Accuracy" OR "Area Under the Curve" OR "AUC" OR "Observer Variation" OR "Interobserver Agreement" OR "Accuracy" OR "Agreement" OR "Concordance") " | 8.282.553 |
|  | **#5** | #1 and #2 and #3 and #4 | **260** |

**Table 3. Bibliographic search strategy in Web of Science**

| **Database** | **WoS** ( [https://www-webofscience-com](https://www-webofscience-com.wdg.biblio.udg.mx:8443/wos/woscc/basic-search) )  **Search date:** Inception - September 20, 2025 | | **Results** |
| --- | --- | --- | --- |
| **Strategy** | **#1** | TS= ("artificial intelligence" OR "machine learning" OR "deep learning") | 1,509,099 |
|  | **#2** | TS= ("orthodontics" OR "malocclusion" OR "cephalometry") | 37,769 |
|  | **#3** | TS= ("diagnosis" OR "computer-assisted diagnosis" OR "cephalometric") | 2,423,209 |
|  | **#4** | TS= ("sensitivity and specificity" OR "area under curve" OR "observer variation" OR "accuracy" OR "agreement" OR "concordance") | 4,094,805 |
|  | **#5** | #1 and #2 and #3 and #4 | **173** |

**Table 4. Bibliographic search strategy in EBSCO**

| **Database** | **EBSCO** ( <https://researchs.uamanizales.elogim.com> )  **Search date:** Inception - September 20, 2025 | | **Results** |
| --- | --- | --- | --- |
| **Strategy** | **#1** | TI ("artificial intelligence" OR "machine learning" OR "deep learning") OR AB ("artificial intelligence" OR "machine learning" OR "deep learning") | 370,593 |
|  | **#2** | TI ("orthodontics" OR "malocclusion" OR "cephalometry") OR AB ("orthodontics" OR "malocclusion" OR "cephalometry") | 16,103 |
|  | **#3** | TI ("diagnosis" OR "computer-assisted diagnosis" OR "cephalometric") OR AB ("diagnosis" OR "computer-assisted diagnosis" OR "cephalometric") | 180,687 |
|  | **#4** | TI ("sensitivity and specificity" OR "area under curve" OR "observer variation" OR "accuracy" OR "agreement" OR "concordance")  OR AB ("sensitivity and specificity" OR "area under curve" OR "observer variation" OR "accuracy" OR "agreement" OR "concordance") | 1,457,734 |
|  | **#5** | #1 and #2 and #3 and #4 | **51** |

**Table 5. Bibliographic search strategy in Scielo**

| **Database** | **SCIELO** (<https://searchscielo.uamanizales.elogim.com> )  **Search date:** Inception - September 20, 2025 | | **Results** |
| --- | --- | --- | --- |
| **Strategy** | **#1** | "artificial intelligence" OR "machine learning" OR "deep learning" | 3.226 |
|  | **#2** | "orthodontics" OR "malocclusion" OR "cephalometry" | 3.017 |
|  | **#3** | "diagnosis" OR "computer-assisted diagnosis" OR "cephalometric" | 67.841 |
|  | **#4** | "sensitivity and specificity" OR "area under curve" OR "observer variation" OR "accuracy" OR "agreement" OR "concordance" | 24.712 |
|  | **#5** | #1 AND #2 AND #3 AND #4 | **4** |

**Table 6. Bibliographic search strategy in IEEE**

| **Database** | **IEEE** (<https://ieeexplore.ieee.org/>)  **Search date:** Inception - September 20, 2025 | | **Results** |
| --- | --- | --- | --- |
| **Strategy** | **#1** | ("artificial intelligence" OR "machine learning" OR "deep learning") | 668.633 |
|  | **#2** | ("orthodontics" OR "malocclusion" OR "cephalometry") | 445 |
|  | **#3** | ("diagnosis" OR "computer-assisted diagnosis" OR "cephalometric") | 131.828 |
|  | **#4** | ("sensitivity and specificity" OR "area under curve" OR "observer variation" OR "accuracy" OR "agreement" OR "concordance") | 817.947 |
|  | **#5** | #1 AND #2 AND #3 AND #4 | **40** |

*Full electronic search strategies for PubMed/MEDLINE, Scopus, Web of Science, IEEE Xplore, EBSCO, and SciELO.*

**Table 7. Bibliographic search strategy in GOOGLE SCHOLAR (citation chasing only; not counted as database yield)**

| **Database** | **GOOGLE SCHOLAR** (<https://scholar.google.com/>)  **Search date:** Inception - September 20, 2025 | | **Results** |
| --- | --- | --- | --- |
| **Strategy** | **#1** | "artificial intelligence" OR "machine learning" OR "deep learning" | 17.800 |
|  | **#2** | "orthodontics" OR "malocclusion" OR "cephalometry" | 588.000 |
|  | **#3** | ("diagnosis" OR "computer-assisted diagnosis" OR "cephalometric diagnosis" OR "cephalometric analysis") | 5.910.000 |
|  | **#4** | ("sensitivity and specificity" OR "area under the curve" OR "AUC" OR "observer variation" OR "diagnostic accuracy" OR "agreement" OR "interobserver concordance" OR "intraobserver concordance") | 6.080.000 |
|  | **#5** | #1 AND #2 AND #3 AND #4 | **6.480** |

**Google Scholar:** 100 records inspected; 0 unique eligible.
