# Supplementary material for: Parameter-level comparison of artificial intelligence and manual cephalometric measurements: a systematic review and meta-analysis
Source: Front Dent Med. 2026 Aug 21;7:1820984. doi: 10.3389/fdmed.2026.1820984 (PMC13541505; doi:10.3389/fdmed.2026.1820984)
Supplement: Supplementary file 2 [file Supplementaryfile10.docx]

Supplementary Material 10 – Narrative Summary of Pooled Effects and Outcome-level GRADE Certainty

| Parameter group | Outcome relative to manual tracing | Certainty of evidence (GRADE) |
| --- | --- | --- |
| ANB | Small statistically significance difference favouring AI (g = 0.28; 95% CI 0.03–0.53); clinical relevance remains uncertain | Low |
| SNA, SNB, U1–NA, L1–NB, IMPA, U1–SN, Interincisal, L1–MeGo, SN–MP, Wits, Gonial | No statistically significant difference or inconclusive findings depending on the outcome; see Supplementary Material 8 | Moderate to Low |
| FMA, SUM–MAN | Inconclusive because of substantial heterogeneity and imprecision | Low |

Note: This supplementary table provides a concise narrative summary of the pooled quantitative findings together with the corresponding outcome-level GRADE certainty assessments. It is intended as an overview of the evidence and should not be interpreted as a formal GRADE Summary of Findings (SoF) table.

- *Certainty judgments (GRADE) triangulate risk of bias, inconsistency (I²), imprecision, indirectness, and small-study/publication bias; see Supplementary Material 8 for the complete outcome-level certainty assessments.*
- *Exploratory Egger's regression analyzes were performed for all outcomes solely for completeness and transparency. Because all outcome-specific meta-analyses included fewer than 10 studies, these exploratory analyzes should not be interpreted as formal assessments of publication bias.*
- *Effect sizes are expressed as Hedges' g unless otherwise specified. Positive values favor AI-derived measurements.*
