# Supplementary material for: Parameter-level comparison of artificial intelligence and manual cephalometric measurements: a systematic review and meta-analysis
Source: Front Dent Med. 2026 Aug 21;7:1820984. doi: 10.3389/fdmed.2026.1820984 (PMC13541505; doi:10.3389/fdmed.2026.1820984)
Supplement: Supplementary file 3 [file Supplementaryfile2.docx]

Supplementary Material 2 – List of Studies Excluded After Full-Text Review

| Author | Year | Reason for exclusion |
| --- | --- | --- |
| Jiang et al. [1] | 2023 | Comparator not based on human orthodontist assessment |
| O’Friel et al. [2] | 2024 |  |
| Gonca et al. [3] | 2024 |  |
| Lee et al. [4] | 2020 |  |
| Weingart et al. [5] | 2023 |  |
| Dot et al. [6] | 2022 |  |
| Tao et al. [7] | 2023 |  |
| Jiang et al. [8] | 2025 |  |
| Vaughan et al. [9] | 2025 |  |
| Le et al. [10] | 2022 |  |
| Moreno & Gebeile-Chauty [11] | 2022 |  |
| Thanathornwong [12] | 2018 |  |
| Neeraja & Jani [13] | 2023 |  |

*List of full-text articles excluded after detailed assessment, with reasons for exclusion (mainly absence of a human or orthodontic specialist comparator).*

**References**

1. Jiang F, Guo Y, Yang C, Zhou Y, Lin Y, Cheng F, et al. Artificial intelligence system for automated landmark localization and analysis of cephalometry. Dentomaxillofac Radiol. 2023;52(1):20220081. doi: 10.1259/dmfr.20220081
2. O’Friel K, Chapple A, Ballard R, Armbruster P. Assessing AudaxCeph®’s cephalometric tracing technology versus a semi-automated approach for analyzing severe Class II and Class III skeletons. Int Orthod. 2024;22(4):100926. doi: 10.1016/j.ortho.2024.100926
3. Gonca, M., Bayrakdar, İ.Ş. & Çelik, Ö. Does the FARNet neural network algorithm accurately identify Posteroanterior cephalometric landmarks? BMC Med Imaging 24, 294 (2024). doi: 10.1186/s12880-024-01478-z
4. Lee, JH., Yu, HJ., Kim, Mj. et al. Automated cephalometric landmark detection with confidence regions using Bayesian convolutional neural networks. BMC Oral Health 20, 270 (2020). doi: 10.1186/s12903-020-01256-7
5. Weingart JV, Schlager S, Metzger MC, Brandenburg LS, Hein A, Schmelzeisen R, et al. Automated detection of cephalometric landmarks using deep neural patchworks. Dentomaxillofac Radiol. 2023;52(6):20230059. doi: 10.1259/dmfr.20230059
6. Dot G, Schouman T, Chang S, Rafflenbeul F, Kerbrat A, Rouch P, et al. Automatic 3-dimensional cephalometric landmarking via deep learning. J Dent Res. 2022;101(11):1380–7. doi: 10.1177/00220345221112333
7. Tao L, Li M, Zhang X, Cheng M, Yang Y, Fu Y, et al. Automatic craniomaxillofacial landmarks detection in CT images of individuals with dentomaxillofacial deformities by a two-stage deep learning model. BMC Oral Health. 2023;23(1):876. doi: 10.1186/s12903-023-03446-5
8. Jiang Y, Jiang C, Shi B, Wu Y, Xing S, Liang H, et al. Automatic identification of hard and soft tissue landmarks in cone-beam computed tomography via deep learning with diversity datasets: a methodological study. BMC Oral Health 2025;25(1):505. doi: 10.1186/s12903-025-05831-8
9. Vaughan M, Mheissen S, Cobourne M, Ahmed F. Diagnostic accuracy of artificial intelligence for dental and occlusal parameters using standardized clinical photographs. Am J Orthod Dentofacial Orthop. 2025;167(6):733–40. doi: 10.1016/j.ajodo.2025.01.017
10. Le VNT, Kang J, Oh I-S, Kim J-G, Yang Y-M, Lee D-W. Effectiveness of human-artificial intelligence collaboration in cephalometric landmark detection. J Pers Med. 2022;12(3):387. doi: 10.3390/jpm12030387
11. Moreno M, Gebeile-Chauty S. Comparative study of two software for the detection of cephalometric landmarks by artificial intelligence. Orthod Fr. 2022;93(1):41–61. doi: 10.1684/orthodfr.2022.73
12. Thanathornwong B. Bayesian-based decision support system for assessing the needs for orthodontic treatment. Healthc Inform Res. 2018;24(1):22–8.  [doi: 10.4258/hir.2018.24.1.22](http://dx.doi.org/10.4258/hir.2018.24.1.22)
13. Neeraja R, Jani Anbarasi L. CephXNet: A deep convolutional squeeze-and-excitation model for landmark prediction on lateral cephalograms. IEEE Access. 2023;11:90780–800. doi: 10.1109/access.2023.3307636
