# Supplementary material for: Parameter-level comparison of artificial intelligence and manual cephalometric measurements: a systematic review and meta-analysis
Source: Front Dent Med. 2026 Aug 21;7:1820984. doi: 10.3389/fdmed.2026.1820984 (PMC13541505; doi:10.3389/fdmed.2026.1820984)
Supplement: Supplementary file 4 [file Supplementaryfile3.docx]

Supplementary Material 3 – Data Extraction Matrix

| Authors | Year | Country | Study design | Population | Sample size | Age | AI system | Groups compared | Radiographs | Reference standard | Outcomes measured | Clinical threshold | Main findings |
| --- | --- | --- | --- | --- | --- | --- | --- | --- | --- | --- | --- | --- | --- |
| Panesar et al. | 2023 | United States | Analytical observational | Adults with skeletal and dental Class I relationships | 30 patients; NR | >18 years; NR | CEFBOT (RadioCef v3), convolutional neural network (CNN) trained with >250,000 images | Orthodontist without AI, resident without AI, general dentist without AI, dental student without AI, orthodontist with AI, resident with AI, general dentist with AI, dental student with AI, AI alone | 30 radiographs traced twice | Orthodontist and radiologist with 25 years of experience; in case of disagreement, the point was placed at the midpoint between both tracings | ICC (intraclass correlation coefficient), MAE (mean absolute error) in mm or degrees | MAE < 2 mm or degrees was considered clinically acceptable | AI significantly improves the precision and accuracy of cephalometric analysis, especially for less experienced examiners. |
| Koz et al. | 2025 | Turkey | Cross-sectional | Patients requiring orthodontic treatment | 300 lateral cephalograms | Mean age: 20.5 ± 5.2 years | YOLOv3 deep learning algorithm for landmark detection | AI vs. manual tracing by orthodontists | One cephalogram per patient | Experienced orthodontist (gold standard) | Accuracy (mm), success detection rate (%) | < 2 mm considered clinically acceptable | AI demonstrated comparable accuracy to manual tracing, with faster processing time. |
| Mercier et al. | 2024 | Spain and international collaboration with Italy and France | Retrospective analytical | Orthodontic patients from clinical database | 120 cephalograms | 12–35 years | Customized CNN model trained on annotated images | AI vs. two experienced orthodontists | One cephalogram per patient | Consensus between two orthodontists | MAE (mm), detection success rate (%) | < 1.5 mm acceptable | AI achieved high agreement with experts, reducing variability in landmark identification. |
| O'Friel et al. | 2024 | United States | Analytical observational | Adults with malocclusion | 80 cephalograms | 18–40 years | Faster R-CNN algorithm integrated into cephalometric software | AI vs. orthodontist | One cephalogram per patient | Orthodontist with >15 years of experience | ICC, MAE (mm) | MAE ≤ 2 mm acceptable | AI performed similarly to expert tracings, improving efficiency. |
| Menezes et al. | 2023 | Brazil | Cross-sectional analytical | Orthodontic patients with Class II malocclusion | 200 cephalograms | Mean age: 16.8 ± 3.1 years | Deep learning model based on ResNet architecture | AI vs. manual tracing by orthodontist | One cephalogram per patient | Senior orthodontist | MAE (mm), time required | < 2 mm acceptable | AI reduced tracing time significantly while maintaining accuracy comparable to manual methods. |
| Bulatova et al. | 2021 | United States | Cross-sectional | Orthodontic patients | 150 cephalograms | 14–25 years | VGGNet-based CNN for automated landmark detection | AI vs. orthodontist | One cephalogram per patient | Orthodontist with >10 years of experience | MAE (mm), detection rate (%) | < 2 mm acceptable | AI achieved accuracy close to that of an expert orthodontist and reduced analysis time by more than half. |
| Kunz et al. | 2025 | Germany | Analytical observational | Mixed dentition patients | 90 cephalograms | 8–12 years | YOLOv4 algorithm | AI vs. orthodontist | One cephalogram per patient | Orthodontist with >15 years of experience | MAE (mm), precision (%) | < 2 mm acceptable | AI demonstrated reliable landmark identification even in mixed dentition cases. |
| Bao et al. | 2023 | China | Retrospective | Orthodontic patients | 85 cephalograms | Mixed and permanent dentition | Planmeca Romexis 6.2 | AI vs. computer-assisted manual tracing | One cephalogram per patient | Manual tracing performed by trained orthodontists | ICC, MRE, SDR, Bland-Altman analysis | 2 mm or 2° acceptable | AI produced clinically acceptable cephalometric measurements with high agreement to manual analysis. |
| Blum et al. | 2023 | Germany | Cross-sectional | Orthodontic patients with craniofacial anomalies | 75 cephalograms | 10–20 years | ResNet-50 CNN | AI vs. orthodontist | One cephalogram per patient | Orthodontist with craniofacial expertise | MAE (mm), detection rate (%) | < 2 mm acceptable | AI maintained high accuracy despite anatomical variations. |
| Mahto et al. | 2022 | Nepal | Analytical observational | Adults with Class III malocclusion | 60 cephalograms | 18–35 years | Faster R-CNN | AI vs. orthodontist | One cephalogram per patient | Orthodontist with >20 years of experience | ICC, MAE (mm) | < 2 mm acceptable | AI performance was comparable to manual tracing, with substantial time savings. |
| Park et al. | 2024 | Belgium | Cross-sectional | Orthodontic patients | 110 cephalograms | 12–25 years | Inception-v3 CNN | AI vs. orthodontist | One cephalogram per patient | Orthodontist with >12 years of experience | MAE (mm), detection success (%) | < 2 mm acceptable | AI showed high agreement with expert tracings and improved workflow efficiency. |
| Katyal et al. | 2022 | India | Retrospective analytical | Orthodontic patients | 140 cephalograms | 15–30 years | U-Net deep learning model for segmentation and landmark detection | AI vs. orthodontist | One cephalogram per patient | Orthodontist with >10 years of experience | MAE (mm), ICC | < 2 mm acceptable | AI provided consistent results with minimal deviation from expert tracings. |
| Lee et al. | 2023 | Korea | Cross-sectional | Orthodontic patients with asymmetry | 85 cephalograms | 14–28 years | CNN with transfer learning from ImageNet | AI vs. orthodontist | One cephalogram per patient | Orthodontist specialized in asymmetry cases | MAE (mm), detection rate (%) | < 2 mm acceptable | AI handled asymmetrical structures accurately, matching expert performance. |
| Khabadze et al. | 2024 | Russia | Analytical observational | Patients undergoing orthognathic surgery | 50 cephalograms | 18–40 years | YOLOv5-based detection system | AI vs. orthodontist | One cephalogram per patient | Orthodontist with >15 years of surgical planning experience | MAE (mm), ICC | < 2 mm acceptable | AI achieved similar accuracy to manual tracings, reducing analysis time. |
| Kim et al. | 2024 | South Korea | Cross-sectional | Orthodontic patients | 180 cephalograms | 13–29 years | DenseNet-based CNN | AI vs. orthodontist | One cephalogram per patient | Experienced orthodontist | MAE (mm), detection rate (%) | < 2 mm acceptable | AI significantly reduced tracing time without compromising accuracy. |
| Kim et al. | 2021 | South Korea | Retrospective analytical | Orthodontic patients with skeletal discrepancies | 95 cephalograms | 16–32 years | CNN model with multi-scale feature extraction | AI vs. orthodontist | One cephalogram per patient | Senior orthodontist | MAE (mm), ICC | < 2 mm acceptable | AI matched expert accuracy and improved repeatability of landmark placement. |
| Ye et al. | 2023 | China | Cross-sectional | Orthodontic patients | 210 cephalograms | 15–27 years | EfficientNet-based CNN | AI vs. orthodontist | One cephalogram per patient | Orthodontist with >10 years of experience | MAE (mm), detection success (%) | < 2 mm acceptable | AI provided results comparable to expert tracings with reduced analysis time. |
| Bor et al. | 2024 | Turkey | Analytical observational | Orthodontic patients with Class II division 2 malocclusion | 70 cephalograms | 14–25 years | ResNeXt deep learning architecture | AI vs. orthodontist | One cephalogram per patient | Orthodontist with >15 years of experience | MAE (mm), ICC | < 2 mm acceptable | AI delivered precise landmark identification in complex malocclusions. |
| Jeon et al. | 2021 | South Korea | Cross-sectional analytical | Orthodontic patients in mixed dentition | 65 cephalograms | 8–12 years | YOLOv3 with custom training dataset | AI vs. orthodontist | One cephalogram per patient | Orthodontist with pediatric experience | MAE (mm), detection rate (%) | < 2 mm acceptable | AI maintained high accuracy despite challenges in mixed dentition cases. |
| Zhang et al. | 2024 | China | Retrospective | Orthodontic patients from academic clinic | 160 cephalograms | 12–26 years | Hybrid CNN-LSTM model | AI vs. orthodontist | One cephalogram per patient | Senior orthodontist | MAE (mm), ICC | < 2 mm acceptable | AI improved landmark detection consistency over manual methods. |
| Mehra et al. | 2024 | India | Cross-sectional | Orthodontic patients with cleft lip and palate | 55 cephalograms | 10–22 years | Customized CNN trained on cleft-specific dataset | AI vs. orthodontist | One cephalogram per patient | Orthodontist specialized in craniofacial anomalies | MAE (mm), detection rate (%) | < 2 mm acceptable | AI maintained accuracy in cases with altered anatomy, similar to expert performance. |
| Ramadan et al. | 2022 | Saudi Arabia | Analytical observational | Orthodontic patients with severe crowding | 100 cephalograms | 14–28 years | YOLOv5 with enhanced feature pyramid network | AI vs. orthodontist | One cephalogram per patient | Orthodontist with >12 years of experience | MAE (mm), ICC | < 2 mm acceptable | AI achieved high agreement with manual tracings while reducing the time required for analysis. |

*Structured extraction table summarizing author, year, country, sample size, population, AI model, comparator, outcomes, and validation method.*
