# Supplementary material for: Parameter-level comparison of artificial intelligence and manual cephalometric measurements: a systematic review and meta-analysis
Source: Front Dent Med. 2026 Aug 21;7:1820984. doi: 10.3389/fdmed.2026.1820984 (PMC13541505; doi:10.3389/fdmed.2026.1820984)
Supplement: Supplementary file 5 [file Supplementaryfile4.docx]

Supplementary Material 4 - Risk of Bias Using QUADAS-2

| Study | Risk of bias | | | | Concerns regarding applicability | | | |
| --- | --- | --- | --- | --- | --- | --- | --- | --- |
|  | Patient Selection | Index Test | Reference Standard | Flow and Timing | Patient Selection | Index Test | Reference Standard | Flow and Timing |
| Panesar et al. [1] | Low | Low | Low | Low | Low | Low | Low | - |
| Koz et al. [2] | Low | Unclear | Low | Low | Unclear | High | High | High |
| Mercier et al. [3] | Unclear | Low | Low | Low | Low | Low | High | High |
| O'Friel et al. [4] | Low | Unclear | Low | Low | Unclear | High | High | High |
| Menezes et al. [5] | Unclear | Low | High | Low | High | High | Low | High |
| Bulatova et al. [6] | Low | Low | Low | Low | Low | Low | High | High |
| Kunz et al. [7] | Low | Unclear | Low | Low | Unclear | High | High | High |
| Bao et al. [8] | Unclear | Low | Low | Low | Unclear | Low | High | - |
| Blum et al. [9] | Low | Low | Low | Low | Low | Low | High | High |
| Mahto et al. [10] | Low | Low | Unclear | Low | Low | High | High | High |
| Park et al. [11] | Low | Low | Low | Low | Low | Low | High | High |
| Katyal et al. [12] | Low | Low | Low | Low | Low | Low | High | High |
| Lee et al. [13] | Low | Unclear | Low | Low | Low | Low | High | High |
| Khabadze et al. [14] | Low | Unclear | Low | Low | Low | Low | High | High |
| Kim et al. [15] | Low | Low | Low | Low | Low | Low | High | High |
| Kim et al. [16] | Unclear | Low | Low | Low | Low | Low | Low | Low |
| Ye et al. [17] | Unclear | Low | Low | Low | Low | Low | High | High |
| Bor et al. [18] | Low | Unclear | Low | Low | Low | High | High | High |
| Jeon et al. [19] | Low | Low | Low | Low | Low | Low | High | High |
| Zhang et al. [20] | Unclear | Low | Low | Low | Unclear | - | Low | - |
| Mehra et al. [21] | Low | Low | Low | Low | Low | Low | High | High |
| Ramadan et al. [22] | Unclear | Low | Unclear | Low | Unclear | High | Unclear | High |
| *Legend: Low = low risk of bias or low concern regarding applicability; High = high risk of bias or high concern regarding applicability; Unclear = unclear risk of bias or unclear concern regarding applicability.* | | | | | | | | |

*Risk-of-bias and applicability assessment of all included studies using the QUADAS-2 tool.*

**References**

1. Panesar S, Zhao A, Hollensbe E, Wong A, Bhamidipalli SS, Eckert G, et al. Precision and Accuracy Assessment of Cephalometric Analyses Performed by Deep Learning Artificial Intelligence with and without Human Augmentation. Appl Sci. 2023;13(12):6921. doi: 10.3390/app13126921
2. Koz S, Uslu-Akcam O. Artificial Intelligence-Supported and App-Aided Cephalometric Analysis: Which One Can We Trust? Diagnostics. 2025;15(5):559. doi: 10.3390/diagnostics15050559
3. Mercier JP, Rossi C, Sanchez IN, Renovales ID, Sahagún PMP, Templier L. Reliability and accuracy of Artificial intelligence-based software for cephalometric diagnosis. A diagnostic study. BMC Oral Health. 2024;24(1):1309. doi: 10.1186/s12903-024-05097-6
4. O’Friel K, Chapple A, Ballard R, Armbruster P. Assessing AudaxCeph®’s cephalometric tracing technology versus a semi-automated approach for analyzing severe Class II and Class III skeletons. Int Orthod. 2024;22(4):100926. doi: 10.1016/j.ortho.2024.100926
5. Menezes LDS, Silva TP, Lima Dos Santos MA, Hughes MM, Mariano Souza SDR, Leite Ribeiro PM, et al. Assessment of landmark detection in cephalometric radiographs with different conditions of brightness and contrast using the an artificial intelligence software. Dentomaxillofacial Radiol. 2023 ;52(8). doi: 10.1259/dmfr.20230065
6. Bulatova G, Kusnoto B, Grace V, Tsay TP, Avenetti DM, Sanchez FJC. Assessment of automatic cephalometric landmark identification using artificial intelligence. Orthod Craniofac Res. 2021;24(S2):37-42. doi: 10.1111/ocr.12542
7. Kunz F, Stellzig-Eisenhauer A, Widmaier LM, Zeman F, Boldt J. Assessment of the quality of different commercial providers using artificial intelligence for automated cephalometric analysis compared to human orthodontic experts. J Orofac Orthop Fortschritte Kieferorthopädie. 2025;86(3):145-60. doi: 10.1007/s00056-023-00491-1
8. Bao H, Zhang K, Yu C, et al. Evaluating the accuracy of automated cephalometric analysis based on artificial intelligence. BMC Oral Health. 2023;23(1):191 doi:10.1186/s12903-023-02881-8
9. Blum FMS, Möhlhenrich SC, Raith S, Pankert T, Peters F, Wolf M, et al. Evaluation of an artificial intelligence-based algorithm for automated localization of craniofacial landmarks. Clin Oral Investig. mayo de 2023;27(5):2255-65. doi: 10.1007/s00784-023-04978-4
10. Mahto RK, Kafle D, Giri A, Luintel S, Karki A. Evaluation of fully automated cephalometric measurements obtained from web-based artificial intelligence driven platform. BMC Oral Health. 2022 ;22(1). doi: 10.1186/s12903-022-02170-w
11. Park J, Yoon S, Kim H, Kim Y, Lee U, Yu H. Clinical validity and precision of deep learning-based cone-beam computed tomography automatic landmarking algorithm. Imaging Sci Dent. 2024;54(3):240. doi: 10.5624/isd.20240009
12. Katyal D, Balakrishnan N. Evaluation of the accuracy and reliability of WebCeph – An artificial intelligence-based online software. APOS Trends Orthod. 2022;12:271-6. doi: 10.25259/APOS_138_2021.
13. Lee J, Bae SR, Noh HK. Commercial artificial intelligence lateral cephalometric analysis: part 2-effects of human examiners on artificial intelligence performance, a pilot study. J Clin Pediatr Dent. 2023;47(6):130-41 doi: 10.22514/jocpd.2023.087
14. Khabadze Z, Mordanov O, Shilyaeva E. Comparative Analysis of 3D Cephalometry Provided with Artificial Intelligence and Manual Tracing. Diagnostics. 2024;14(22):2524. doi: 10.3390/diagnostics14222524
15. Kim KH, Min JH, Yun J hyun, Baek JH, Song IS, Lee SM, et al. Evaluation of the clinical utility of lateral cephalometry reconstructed from computed tomography extracted by artificial intelligence. J Cranio-Maxillofac Surg. marzo de 2025;53(3):214-21. doi: 10.1016/j.jcms.2024.12.004
16. Kim YH, Park JB, Chang MS, Ryu JJ, Lim WH, Jung SK. Influence of the Depth of the Convolutional Neural Networks on an Artificial Intelligence Model for Diagnosis of Orthognathic Surgery. J Pers Med. 2021;11(5):356. doi: 10.3390/jpm11050356
17. Ye H, Cheng Z, Ungvijanpunya N, Chen W, Cao L, Gou Y. Is automatic cephalometric software using artificial intelligence better than orthodontist experts in landmark identification? BMC Oral Health. 2023;23(1). doi: 10.1186/s12903-023-03188-4
18. Bor S, Ciğerim SÇ, Kotan S. Comparison of AI-assisted cephalometric analysis and orthodontist-performed digital tracing analysis. Prog Orthod. 2024; 25(1). doi: 10.1186/s40510-024-00539-x
19. Jeon S, Lee KC. Comparison of cephalometric measurements between conventional and automatic cephalometric analysis using convolutional neural network. Prog Orthod. 2021;22(1):14. doi: 10.1186/s40510-021-00358-4
20. Zhang H, Liu C, Yang P, Yang S, Yu Q, Liu R. The concept of AI-assisted self-monitoring for skeletal malocclusion. Health Informatics J. 2024;30(3). doi: 10.1177/14604582241274511
21. Mehra P, R N, Anbarasi L J, Ravi V, Al Mazroa A. VGG-16 based Deep Learning Approach for Cephalometric Landmark Detection. Open Public Health J. 2024;17(1). doi: 10.2174/0118749445338097240923063441
22. Ramadan RA, Khedr AY, Yadav K, Alreshidi EJ, Sharif MH, Azar AT, et al. Convolution neural network based automatic localization of landmarks on lateral x-ray images. Multimed Tools Appl. noviembre de 2022;81(26):37403-15. doi: 10.1007/s11042-021-11596-3
