# Supplementary material for: Parameter-level comparison of artificial intelligence and manual cephalometric measurements: a systematic review and meta-analysis
Source: Front Dent Med. 2026 Aug 21;7:1820984. doi: 10.3389/fdmed.2026.1820984 (PMC13541505; doi:10.3389/fdmed.2026.1820984)
Supplement: Supplementary file 6 [file Supplementaryfile5.docx]

Supplementary Material 5 - Funding sources and conflicts of interest

| **Study** | **Funding Source** | **Conflict of Interest** |
| --- | --- | --- |
| Panesar et al. | No external funding | No conflict of interest |
| Senol Koz et al. | No external funding | No conflict of interest |
| Jean-Philippe Mercier et al. | No external funding | No competing interests |
| Katherine O'Friel et al. | No external funding; in-kind support (AudaxCeph® software donated by AudaxCeph® CEO) | No competing interests |
| Dos Santos Menezes L et al. | NR | No conflict of interest |
| Galina Bulatova et al. | NR | No conflict of interest |
| Kunz F et al. | Open Access funding enabled by Projekt DEAL | No conflict of interest |
| Khan EB et al. | No external funding | No conflict of interest |
| Blum FMS et al. | Open Access funding enabled by Projekt DEAL | No competing interests |
| Mahto RK et al. | None | No competing interests |
| Jungeun Park et al. | Korea Medical Device Development Fund grant (Korean government, Project No. RS-2020-KD000002) | No conflict of interest |
| Deepika Katyal et al. | None | No conflict of interest |
| Jaesik Lee et al. | Kyungpook National University Dental Hospital Institute for Dental Research (2022) | No conflict of interest |
| Zurab Khabadze et al. | No external funding | No conflict of interest |
| Kyung-Han Kim et al. | Korea Health Technology R&D Project (KHIDI, Ministry of Health and Welfare, HI23C0162) and Basic Science Research Program (NRF, Ministry of Education, 2021R1I1A1A01059584) | No competing interests |
| Ye-Hyun Kim et al. | No external funding | No conflict of interest |
| Huayu Ye et al. | National Natural Science Foundation of China (NSFC, 81900964); Youth Innovation in Future Medicine, Chongqing Medical University (W0033); New Technology and New Project of Stomatological Hospital of Chongqing Medical University (2022, No.15) | No competing interests |
| Bor et al. | None | No competing interests |
| Sangmin J. et al. | National Research Foundation of Korea (NRF), Ministry of Science and ICT (No. 2020R1F1A1070617; NRF-2017R1D1A1B03032132) | No competing interests |
| Zhang H et al. | Clinical Innovation Project of Army Medical University (No. 2019XLC2014) | No competing interests |
| Mehra P et al. | None | No competing interests |
| Ramadan et al. | NR | No conflict of interest |

*Note: The terms “no conflict of interest” and “no competing interests” are reported as stated in the original publications. “NR” indicates not reported.*

*Summary table presenting the funding sources and conflict of interest declarations of the included studies.*
