# Supplementary material for: Parameter-level comparison of artificial intelligence and manual cephalometric measurements: a systematic review and meta-analysis
Source: Front Dent Med. 2026 Aug 21;7:1820984. doi: 10.3389/fdmed.2026.1820984 (PMC13541505; doi:10.3389/fdmed.2026.1820984)
Supplement: Supplementary file 7 [file Supplementaryfile6.docx]

Supplementary Material 6 – Forest Plots, Funnel Plots and Egger’s Test for All Outcomes

**Figure S1. Meta-analysis of U1–NA (°) comparing artificial intelligence and manual cephalometric tracing. Forest plot, funnel plot, and trim-and-fill analysis with Egger’s test results.**

Egger's test = 0.4179

**Figure S2. Meta-analysis of U1–NA (mm) comparing artificial intelligence and manual cephalometric tracing. Forest plot, funnel plot, and trim-and-fill analysis with Egger’s test results.**

Egger’s test= 0.4453

**Figure S3. Meta-analysis of L1-NB (◦) comparing artificial intelligence and manual cephalometric tracing. Forest plot, funnel plot, and trim-and-fill analysis with Egger’s test results.**

Egger’s test= 0.3287

**Figure S4. Meta-analysis of L1-NB (mm) comparing artificial intelligence and manual cephalometric tracing. Forest plot, funnel plot, and trim-and-fill analysis with Egger’s test results.**

Egger’s test= 0.9278

**Figure S5. Meta-analysis of IMPA (°) comparing artificial intelligence and manual cephalometric tracing. Forest plot, funnel plot, and trim-and-fill analysis with Egger’s test results.**

Egger’s test= 0.5417

**Figure S6. Meta-analysis of U1-SN (°) comparing artificial intelligence and manual cephalometric tracing. Forest plot, funnel plot, and trim-and-fill analysis with Egger’s test results.**

Egger’s test= 0.8350

**Figure S7. Meta-analysis of the Interincisal angle (U1-L1) (°) comparing artificial intelligence and manual cephalometric tracing. Forest plot, funnel plot, and trim-and-fill analysis with Egger’s test results.**

Egger’s test= 0.5083

**Figure S8. Meta-analysis of** **L1-MeGo (°) comparing artificial intelligence and manual cephalometric tracing. Forest plot, funnel plot, and trim-and-fill analysis with Egger’s test results**

Egger’s test= 0.0300

**Figure S9. Meta-analysis of** **SNA (°) comparing artificial intelligence and manual cephalometric tracing. Forest plot, funnel plot, and trim-and-fill analysis with Egger’s test results**

Egger’s test= 0.5279

**Figure S10. Meta-analysis of** **SNB (°) comparing artificial intelligence and manual cephalometric tracing. Forest plot, funnel plot, and trim-and-fill analysis with Egger’s test results**

Egger’s test= 0.4395

**Figure S11. Meta-analysis of** **FMA (°) comparing artificial intelligence and manual cephalometric tracing. Forest plot, funnel plot, and trim-and-fill analysis with Egger’s test results**

Egger’s test= 0.12

**Figure S12. Meta-analysis of** **SN-MP (◦) comparing artificial intelligence and manual cephalometric tracing. Forest plot, funnel plot, and trim-and-fill analysis with Egger’s test results**

Egger’s test= 0.4287

**Figure S13. Meta-analysis of** **ANB (◦) comparing artificial intelligence and manual cephalometric tracing. Forest plot, funnel plot, and trim-and-fill analysis with Egger’s test results**

Egger’s test= 0.2514

**Figure S14. Meta-analysis of** **Witts (mm) comparing artificial intelligence and manual cephalometric tracing. Forest plot, funnel plot, and trim-and-fill analysis with Egger’s test results**

Witts (mm)

Egger’s test= 0.2922

**Figure S15. Meta-analysis of** **Gonial (°) comparing artificial intelligence and manual cephalometric tracing. Forest plot, funnel plot, and trim-and-fill analysis with Egger’s test results**

Egger’s test= 0.3335

**Figure S16. Meta-analysis of** **SUM-MAN (°) comparing artificial intelligence and manual cephalometric tracing. Forest plot, funnel plot, and trim-and-fill analysis with Egger’s test results**

SUM-MAN (° ) o BJORK sum o Bjork-Jarabak Sum

Egger’s test= 0.0222
