# Supplementary material for: Parameter-level comparison of artificial intelligence and manual cephalometric measurements: a systematic review and meta-analysis
Source: Front Dent Med. 2026 Aug 21;7:1820984. doi: 10.3389/fdmed.2026.1820984 (PMC13541505; doi:10.3389/fdmed.2026.1820984)
Supplement: Supplementary file 8 [file Supplementaryfile7.docx]

Supplementary Material 7 – GRADE Evidence Profile and Summary of Findings

*Summary of Findings and Certainty of the Evidence (GRADE) for AI Diagnostic Accuracy in Cephalometric Analysis.*

| **Outcome** | **No. of studies (No. of patients)** | **Study design** | **Factors that may decrease the certainty of the evidence** | | | | | **QoE of test accuracy** |
| --- | --- | --- | --- | --- | --- | --- | --- | --- |
|  |  |  |  |  |  |  |  |  |
|  |  |  | **Risk of bias** | **Indirect evidence** | **Inconsistency** | **Imprecision** | **Publication bias** |  |
| **True positives** | 7 studies 414 patients | Cross-sectional | not serious | not serious | not serious | serious | Strongly suspected publication bias | ⨁⨁◯◯ Low |
|  |  |  |  |  |  |  |  |  |
| **False negatives** |  |  |  |  |  |  |  |  |
|  |  |  |  |  |  |  |  |  |
| **True negatives** | 7 studies 414 patients | corte transversal | not serious | not serious | not serious | serious | Strongly suspected publication bias | ⨁⨁◯◯ Low |
|  |  |  |  |  |  |  |  |  |
| **False positives** |  |  |  |  |  |  |  |  |
|  |  |  |  |  |  |  |  |  |
