# Supplementary material for: Parameter-level comparison of artificial intelligence and manual cephalometric measurements: a systematic review and meta-analysis
Source: Front Dent Med. 2026 Aug 21;7:1820984. doi: 10.3389/fdmed.2026.1820984 (PMC13541505; doi:10.3389/fdmed.2026.1820984)
Supplement: Supplementary file 9 [file Supplementaryfile8.docx]

Supplementary Material 8 - Pooled Outcome Overview

Summary of parameter-level outcomes comparing AI vs manual cephalometric measurements, grouped as: no significant difference, AI advantage, and uncertain/high heterogeneity.

| Outcome group | Parameters included | Summary finding |
| --- | --- | --- |
| No significant difference | SNA, SNB, U1–NA (angle and mm), L1–NB (angle and mm), IMPA, U1–SN, Interincisal angle, L1–MeGo, SN–MP, Wits, Gonial | AI and manual methods yielded similar results; no significant differences found |
| Small AI advantage | ANB | Small but statistically significant difference in favour of AI (g = 0.28; 95% CI: 0.03–0.53) |
| Uncertain / high heterogeneity | FMA, SUM–MAN | Results inconsistent; substantial heterogeneity and potential publication bias |

- *Effect sizes are Hedges’ g unless otherwise specified; positive values please AI. Group labels reflect pooled meta-analytic direction and precision.*
- *Uncertain/high heterogeneity = wide CIs and/or substantial inconsistency precluding firm conclusions.*
- *Abbreviations: ANB, SNA, SNB, IMPA, U1–NA, L1–NB, U1–SN, L1–MeGo, SN–MP, FMA, Wits, Gonial, SUM–MAN.*
- *Exploratory Egger's regression analyzes were performed for all outcome-specific meta-analyses. Because all analyzes included fewer than the recommended 10 studies, these results should be interpreted cautiously and not as formal assessments of publication bias.*
