# Supplementary material for: Parameter-level comparison of artificial intelligence and manual cephalometric measurements: a systematic review and meta-analysis
Source: Front Dent Med. 2026 Aug 21;7:1820984. doi: 10.3389/fdmed.2026.1820984 (PMC13541505; doi:10.3389/fdmed.2026.1820984)
Supplement: Supplementary file 10 [file Supplementaryfile9.docx]

Supplementary Material 9 - Pooled Effects and GRADE Certainty

| Parameter group | Outcome relative to manual tracing | Certainty of evidence (GRADE) |
| --- | --- | --- |
| ANB angle | Small AI superiority (g = 0.28; 95% CI 0.03–0.53) | Low |
| SNA, SNB, IMPA, others | No statistically significant difference | Moderate |
| FMA, SUM–MAN | Uncertain (substantial heterogeneity) | Low |
| U1–NA (mm), SUM–MAN | Possible publication bias | Low |

Note: Outcomes grouped into equivalence, AI superiority, or uncertain results.

- *Certainty judgments (GRADE) triangulate risk of bias, inconsistency (I²), imprecision, indirectness, and small-study/publication bias; see Supplementary Material 8 for full SoF details.*
- *Where noted, small-study/publication bias signals are based on Egger's test (p<0.05) when ≥10 studies were available.*
- *Effect sizes are Hedges’ g unless otherwise specified; positive values please AI. The small ANB difference has uncertain clinical relevance.*
